# Cerebrovascular accidents – Unexpected complication of systemic sclerosis: A scarce case report

**DOI:** 10.1097/MD.0000000000044331

**Published:** 2025-09-05

**Authors:** Mohammad Berro, Kenana Tawashi, Mohammad Sharaf, Lin Ibrahim, Osama Abdulaziz

**Affiliations:** a Faculty of Medicine, Damascus University, Damascus, Syria; b Al Mouwasat University Hospital, Damascus University, Damascus, Syria.

**Keywords:** cardiovascular accident, carotid stenosis, case report, rheumatoid, systemic sclerosis, vascular disease

## Abstract

**Rationale:**

Systemic sclerosis (SS) is an immune-mediated connective disease characterized by skin fibrosis, microvascular damage, and multisystem manifestations. One of the most important processes in connective tissue disorders is vasculitis. The clinical findings can differ when the disease is presented with an antineutrophil cytoplasmic antibody. Macrovascular disease in SS patients is rare, while the heart is the most affected organ in this condition; cerebrovascular involvement is sporadic.

**Patient concerns:**

A 46-year-old male presented with left-sided hemiparesis, accompanied by numbness, tingling, and blurred vision in the right eye. These symptoms lasted several minutes before resolving and had occurred more than 10 times in the last month. The patient was diagnosed with SS, Sjögren’s disease, and hypertension 3 months ago after 15 years of symptoms accompanied by SS. The clinical examination revealed stiffened skin, predominantly on the face and hands, as well as generalized dullness of respiratory sounds. The examination of the nervous system was normal.

**Diagnoses:**

A color Doppler ultrasound of the carotid arteries indicated 90% stenosis of the right internal carotid artery. A multisegment computed tomography scan of the aortic arch, branches, and carotid arteries showed many interruptions in several arteries. Magnetic resonance imaging of the brain showed multiple high-signal foci and plaques, which may indicate the presence of vascular lesions. Therefore, the patient was diagnosed with an ischemic cerebrovascular accident secondary to SS.

**Interventions and outcomes:**

Treatment included aspirin, clopidogrel, atorvastatin calcium, azathioprine, prednisone, and hypertension medications. The patient was referred to the Department of Vascular Surgery for stent placement or bypass surgery. During follow-up, the patient developed 4 additional ischemic strokes similar to the previous episodes.

**Lessons:**

This case highlights a patient with SS who presented with macrovascular stenosis. This unusual presentation underscores the importance of vigilance for macrovascular involvement in SS patients presenting with neurological symptoms.

## 1. Introduction

Systemic sclerosis (SS) is an immune-mediated connective disease characterized by skin fibrosis, microvascular damage, and multisystem manifestations.^[1,2]^ In addition, it can be defined as an abnormal repairing response to tissue injuries. It affects 1/10,000 people around the world and is classified as follows: limited cutaneous SS (involvement of the limbs distal to the knees and elbows with or without face and neck involvement), diffuse cutaneous SS (involvement of the limbs proximal to the knees and elbow), and sine scleroderma (patients with clinical manifestations and positive SS-antibodies without skin involvement).^[3]^ The diagnosis of SS is made depending on the 2013 European League against Rheumatism and American College of Rheumatology classification criteria.^[4]^ The clinical manifestations include skin tightness, itching, musculoskeletal pain, lower limb swelling, muscle weakness, and fatigue in early-stage disease. In addition, we see Raynaud phenomenon (RP), calcinosis, skin thickening, and digital ulcers with gastrointestinal and pulmonary symptoms.^[2,3]^ One of the most important processes in the majority of connective tissue disorders is vasculitis, which results from an inflammatory state in the vascular walls; it can be found in vessels with different sizes and sites. Vasculitis in SS forms a cornerstone manifest in addition to fibrosis and auto-immunity and affects small vessels (microvascular disease). The clinical findings can differ when the SS presented with antineutrophil cytoplasmic antibody-associated vasculitis (these cases form only 7% of all SS patients). Macrovascular disease in SS patients is rare, while the heart is the most affected organ in this condition; cerebrovascular involvement is sporadic.^[5]^ We report here one of the rarest cases in the medical literature, where the patient diagnosed with SS, Sjogren syndrome, and (C antineutrophil cytoplasmic antibody)-associated vasculitis developed cerebral infraction many times because of macrovascular disease, affecting the carotid arteries and their branches.

## 2. Case presentation

**Figure 1.**
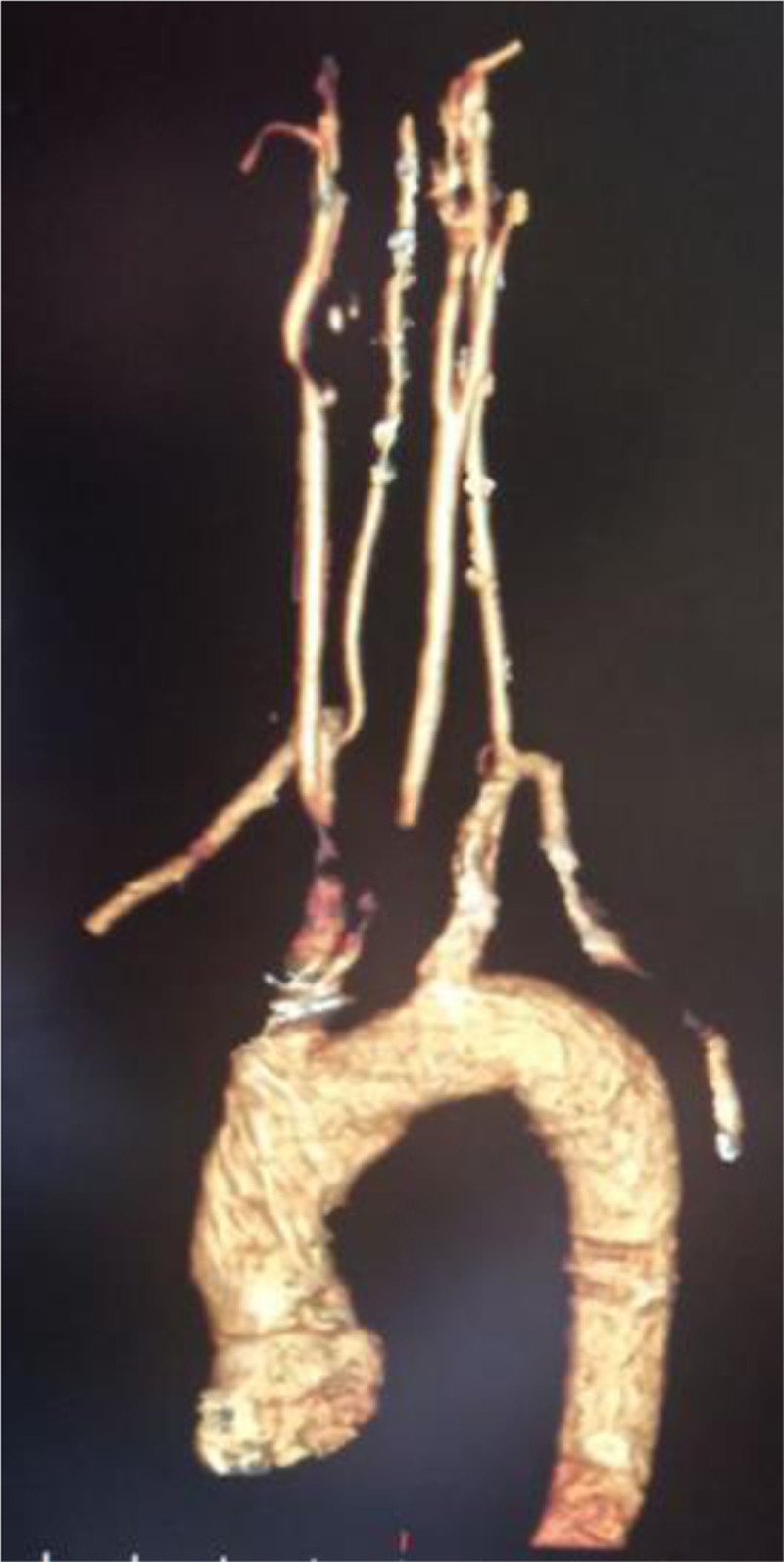
A multisegment CT scan of the aortic arch, its branches, and the carotid arteries showed that the left common carotid artery had a common origin with the brachiocephalic trunk, with acceptable continuity at a distance of 27 mm, followed by an interruption at a distance of 16 mm, and then good recurrence. The right subclavian artery exhibited vascular interruptions 2 cm after its origin spanning 15 mm. The right common carotid artery showed an annular interruption at its beginning followed by recurrence. The right external carotid artery demonstrated 50% annular stenosis at its origin over 5 mm. The right internal carotid artery appeared to be interrupted for 18 mm before recanalization. CT = computed tomography.

**Figure 2.**
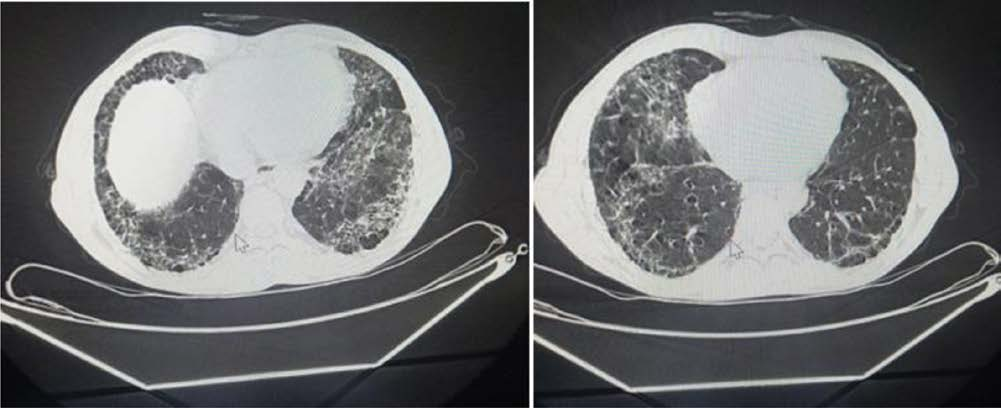
A noncontrast multisegment CT scan of the chest demonstrated reticular densities with honeycomb patterns distributed throughout the lung areas, particularly at the bases and peripheries. CT = computed tomography.

**Figure 3.**
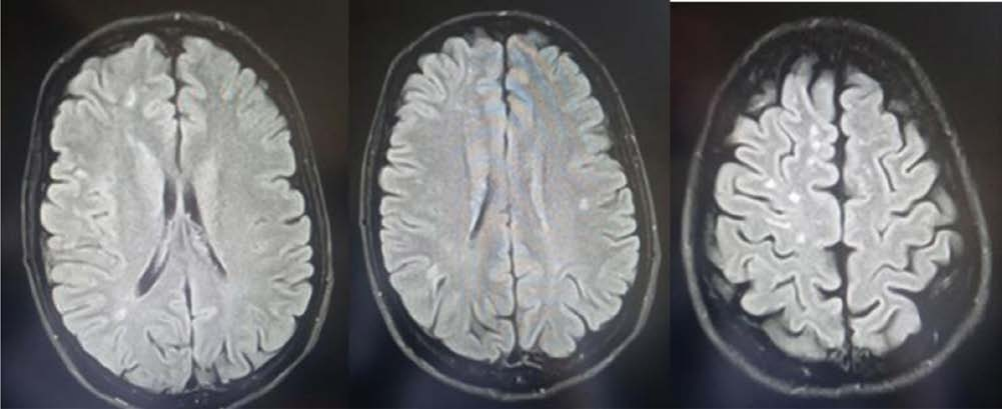
An MRI of the brain with the FLAIR technique showed multiple high-signal foci and plaques in the white matter of the hemispheres, where most of them were located in the peripheral subcortical areas. A small cavity focus was also identified in the context of an old infarction in the right posterior temporal lobe. MRI = magnetic resonance imaging.

A 46-year-old male presented to our department with left-sided hemiparesis, accompanied by numbness, tingling, and blurred vision in the right eye. These symptoms lasted several minutes before resolving and had occurred more than 10 times in the last month. The patient did not use tobacco, alcohol, and illicit drugs. A previous computed tomography (CT) scan revealed no abnormalities. The patient’s medical history included symptoms consistent with RP experienced 15 years ago, which led to necrosis and atrophy at the distal ends of the fingers, particularly in the upper extremities. Three months ago, he consulted a physician and was diagnosed with SS, which later led to the development of pulmonary fibrosis. Additionally, he was diagnosed 1 month ago with secondary Sjögren’s disease and hypertension. The surgical and familial histories were unremarkable. Regarding his medication history, the patient was taking azathioprine and prednisone (10 mg once daily), along with blood pressure control medicines with partial response. Clinical examination revealed stiffened skin predominantly on the face and hands and generalized dullness of respiratory sounds, while the nervous system was normal. The main laboratory results and lipid profile were shown in Table 1. Immunological studies showed the next findings in Table 2. A color Doppler ultrasound of the extremities demonstrated severe spasms in the tibial, radial, and ulnar arteries, while another color Doppler ultrasound of the carotid arteries indicated 90% stenosis of the right internal carotid artery. A multisegment CT scan of the aortic arch, its branches, and the carotid arteries showed that the aortic arch, left subclavian artery, left external carotid artery, left internal carotid artery, left vertebral artery, brachiocephalic trunk, and right vertebral artery were all normal. The left common carotid artery had a common origin with the brachiocephalic trunk, with acceptable continuity at a distance of 27 mm, followed by an interruption at a distance of 16 mm, and then good recurrence. The right subclavian artery exhibited vascular interruptions 2 cm after its origin spanning 15 mm. The right common carotid artery showed an annular interruption at its beginning followed by recurrence. The right external carotid artery demonstrated 50% annular stenosis at its origin over 5 mm. The right internal carotid artery appeared to be interrupted for 18 mm before recanalization (Fig. 1). A chest X-ray revealed increased bronchial vascular markings, bilateral basal alveolar densities, and congestion in the right pulmonary hilum. A noncontrast multisegment CT scan of the chest demonstrated reticular densities with honeycomb patterns distributed throughout the lung areas, particularly at the bases and peripheries, consistent with pulmonary fibrosis (Fig. 2). A magnetic resonance imaging of the brain with the FLAIR technique showed multiple high-signal foci and plaques. These foci were distributed in the white matter of the hemispheres, where most of them were located in the peripheral subcortical areas, without any noticeable enhancement after contrast injection, which may indicate the presence of vascular lesions. A small cavity focus was also identified in the context of an old infarction in the right posterior temporal lobe, with no recent infarct foci currently present. Nasal turbinate hypertrophy with a deviation was also noted (Fig. 3). The patient was referred to the ophthalmology department for an eye consultation. Results showed that the corrected visual acuity in both eyes was 10/10. The pupils were round and reactive, with no relative afferent pupillary defect noted in either eye. The fundus examination was within normal limits, and eye movements were free and normal. There was posterior eyelid margin inflammation in both eyes, with mild conjunctival congestion medially. Inferior corneal punctate epithelial defects and mild nuclear cataracts of the lens were also noted. The tear film was also foamy, and the tear film breakup time was 6 seconds. The patient was diagnosed with an ischemic cerebrovascular accident secondary to SS. Treatment included aspirin (81 mg daily), clopidogrel (75 mg daily), atorvastatin calcium (20 mg daily), and the rest of the treatment was the same as before. The patient was referred to the Department of Vascular Surgery for stent placement or bypass surgery. He was then discharged from the hospital while continuing the aforementioned medications. During follow-up, the patient developed 4 additional ischemic strokes similar to the previous episodes during a month. However, due to financial constraints, the patient was unable to complete follow-up care with doctors or the hospital.

**Table 1:** Laboratory values and lipid profile .

Red blood cell [RBC] count	4.74 × 10^6^/mm^3^
White blood cell [WBC] count	8.6 × 10^3^/mm^3^
Hemoglobin	14.0 g/dL
Mean corpuscular volume [MCV]	86.5 fL
Mean corpuscular hemoglobin [MCH]	29.5 pg
Hematocrit [HCT]	41%
Platelets	232 × 10^3^/mm^3^
Lymphocytes	32%
Neutrophils	67%
Erythrocyte sedimentation rate [ESR]	63 mm/h
C-reactive protein [CRP]	<0.30 mg/dL
Creatinine	0.68 mg/dL
Urea	31 U/L
Fasting blood glucose	96 mg/dL
LDL-C	54 mg/dL
HDL-C	35 mg/dL
Total cholesterol	97 mg/dL
Triglycerides	114 mg/dL

**Table 2:** Immunological studies.

Anticardiolipin IgG	1.5 U/mL; positive
Anti-SS.A [Ro] IgG	210 U/mL; positive
Anticardiolipin IgM	1.2 U/mL
Anti-SS.B [La] IgG	5 U/mL
Beta-2-glycoprotein IgM	7.3 U/L; negative
Beta-2-glycoprotein IgG	5.8 U/L; negative
Anti-centromere antibodies	9; negative
Anti-Scl-70 antibodies	127; positive
Microalbuminuria	Negative
Hepatitis B surface [HBs] antigen	Negative
Hepatitis C virus [HCV] antibody	Negative
Perinuclear antineutrophil cytoplasmic antibody [ANCA-P]	1.3 U/mL; negative
Cytoplasmic ANCA proteinase 3 antibody [ANCA-C]	6.2 U/mL; positive
Antinuclear antibody [ANA] screening test:	
ELISA	225; positive
IgG: homogeneous pattern	1:320

## 3. Discussion

SS is a rare, immune-mediated disease with high mortality, surpassing other rheumatic diseases despite improved survival rates for some, such as those with diffuse cutaneous SS. Its rarity and delayed diagnosis contribute to patient burden, along with the prevalence of distressing information online, which can heighten anxiety for patients and their families.^[3]^ Skin thickening of the fingers extending proximal to the metacarpophalangeal joints is considered sufficient for classifying a patient as having SS. In the absence of this criterion, 7 additional items are evaluated, each with different weights: finger skin thickening, fingertip lesions, telangiectasia, abnormal nail fold capillaries, interstitial lung disease or pulmonary arterial hypertension, RP, and autoantibodies associated with SS.^[4]^ Some of the major complications of SS are digital vasculopathy, gastrointestinal complications, lung fibrosis, pulmonary hypertension, cardiac fibrosis, scleroderma renal crisis, digital contractures, calcinosis, and acro-osteolysis. Treatments for these major complications are diverse.^[3]^ Our patient has been suffering from skin sclerosis, symptoms consistent with RP for 15 years, and lung fibrosis, confirming the diagnosis of SS. In limited SS, skin involvement is restricted to the face, neck, and areas distal to the elbows and knees. In diffuse SS, it extends proximally to include the upper arms, thighs, and/or trunk.^[4]^ In our case, the patient is suffering from left hemiparesis with numbness and tingling; signs of skin tightening, most severe in the face and hands; and erosions at the fingertips of all 4 limbs, most severe in the upper limbs. SS is characterized by microvascular damage due to fibrosis and endothelial cell dysfunction, but Macrovascular disease is considered very rare in SS, which is the case in our patient, making this case exceptional and clinically significant.^[5]^ In addition to that, our patient has developed multiple CVA, which is rare in SS patients. A study from the University of Pennsylvania tracked 2080 SS patients and found that 36 individuals (~1.7%) were diagnosed with CVA after their SS diagnosis.^[6]^ Scholars generally agree that SS can promote premature atherosclerosis and accelerate the progression of arterial stenosis.^[7]^ A meta-analysis showed that the prevalence of atherosclerosis was increased in all vessels in patients with SS,^[8]^ and SS is associated with an increased risk of developing coronary heart disease and stroke.^[7,9]^ In addition, Caimmi et al discovered that the prevalence of carotid plaques in patients with SS was common, but only a small proportion of patients had significant stenosis.^[10]^ Contradicting data have been reported regarding the prevalence of cerebrovascular involvement in SS, but the current opinion is that it is deficient. This case highlights a rare and atypical progression of SS, where carotid artery involvement, rather than the commonly affected microvasculature, led to recurrent ischemic strokes. However, in this case, the combination of SS and chronic hypertension was the primary contributor to the development of stenosis and occlusion in the carotid arteries, a phenomenon documented in only a few cases in the medical literature.^[11]^ The strokes in this patient represent a severe complication of cerebral ischemia secondary to carotid artery stenosis. The transient ischemic attacks preceding the initial stroke, presenting as temporary hemiparesis and visual disturbances, are warning signs of significant vascular lesions, as supported by studies showing increased stroke risk in SS patients with vascular involvement.^[12]^ Pulmonary fibrosis may contribute to pulmonary hypertension, increasing circulatory strain and exacerbating arterial injury. Imaging findings confirmed widespread fibrosis in both lungs, supporting a causal link. In our case, SS was managed with prednisone and azathioprine, following the British Society for Rheumatology and British Health Professionals in Rheumatology guidelines, which recommend the use of glucocorticoids and immunosuppressive agents for treating SS.^[13]^ According to publications from the American Heart Association, carotid artery stenosis was managed with statins, specifically atorvastatin calcium.^[14]^ A study in the American Journal of Neuroradiology also emphasizes the effectiveness of dual antiplatelet therapy, combining aspirin and clopidogrel, in treating this condition.^[15]^ Studies indicate that large vessel involvement in SS is rare but not impossible. According to a report published in the *Journal of Rheumatology* (2021), the involvement of large vessels such as the carotid or aorta can result from chronic inflammation and altered hemodynamics due to systemic fibrosis.

## 4. Conclusion

This case highlights a patient with SS who presented with both microvascular and macrovascular stenosis, a condition that is considered rare. This unusual presentation underscores the importance of vigilance for macrovascular involvement in SS patients presenting with neurological symptoms, as early identification and targeted management could potentially mitigate severe complications. When patients are diagnosed with SS, the cerebral, coronary, and aortic arteries should be examined for stenosis that could lead to serious complications. Early detection, diagnosis, and treatment are essential to reducing disease progression and preventing complications. Further studies are needed to understand better the pathological mechanisms of SS, its role in vasoconstriction, and its effects on the brain.

## Acknowledgments

The authors would like to thank the patient for consenting to publish her case.

## Author contributions

**Writing – original draft:** Mohammad Berro, Kenana Tawashi, Mohammad Sharaf.

**Writing – review & editing:** Lin Ibrahim, Osama Abdulaziz.
